# Disability in cerebellar ataxia syndromes is linked to cortical degeneration

**DOI:** 10.1007/s00415-023-11859-z

**Published:** 2023-07-22

**Authors:** Julian Conrad, Anna Huppert, Ria Maxine Ruehl, Max Wuehr, Roman Schniepp, Peter zu Eulenburg

**Affiliations:** 1 German Center for Vertigo and Balance Disorders and Department of Neurology, Munich University Hospital, LMU Munich, Marchioninistr. 15, 81377 Munich, Germany; 2 German Center for Vertigo and Balance Disorders (DSGZ), LMU Munich, Munich, Germany; 3 Division for Neurodegenerative Diseases, Department of Neurology, Universitaetsmedizin Mannheim, University of Heidelberg, Theodor-Kutzer Ufer 1-3, 68167 Mannheim, Germany; 4 Institute for Neuroradiology LMU Munich, Munich, Germany; 5 Graduate School of Systemic Neurosciences-GSN, LMU Munich, Munich, Germany

**Keywords:** Voxel-based morphometry, Surface-based morphometry, Cerebellar, Downbeat nystagmus, Spinocerebellar ataxia

## Abstract

**Objective:**

We aimed to relate clinical measures of disability in chronic cerebellar degeneration to structural whole-brain changes using voxel-based and surface-based morphometry (*vbm and sbm*). We were particularly interested in remote effects of cerebellar degeneration in the cerebral cortex.

**Methods:**

We recruited 30 patients with cerebellar degeneration of different aetiologies (downbeat nystagmus syndrome, DBN *n* = 14, spinocerebellar ataxia, SCA *n* = 9, sporadic adult late-onset ataxia, SAOA *n* = 7). All patients were thoroughly characterised in the motor, cognitive, vestibular and ocular–motor domains. *Vbm* and *sbm* were used to evaluate structural differences between cerebellar degeneration patients and a group of healthy age- and gender-matched volunteers. Linear regression models were used to correlate functional measures of disease progression and postural stability with whole brain volumetry.

**Results:**

Patients with SCA and SAOA showed widespread volume loss in the cerebellar hemispheres and less prominently in the vermis. Patients with DBN showed a distinct pattern of grey matter volume (GMV) loss that was restricted to the vestibular and ocular–motor representations in lobules IX, X and V–VII. Falls were associated with brainstem white matter volume. *VBM* and *SBM* linear regression models revealed associations between severity of ataxic symptoms, cognitive performance and preferred gait velocity. This included extra-cerebellar (sub-)cortical hubs of the motor and locomotion network (putamen, caudate, thalamus, primary motor cortex, prefrontal cortex) and multisensory areas involved in spatial navigation and cognition.

**Conclusion:**

Functional disability in multiple domains was associated with structural changes in the cerebral cortex.

## Introduction

Patients with chronic cerebellar degeneration typically present with dys-coordinated limb movements, impairments of gait stability, vestibular/ocular–motor symptoms, dysarthria and dysphagia. “Non-cerebellar” symptoms, such as neuropathy, extrapyramidal, pyramidal signs and cognitive impairments, are also present in both hereditary, genetically confirmed ataxias, and sporadic adult-onset ataxias (SAOA) [15, 27, 38]. These phenomenological findings are supported by tracer and neuroimaging studies on the interconnections of cerebellar and cerebral cortices [2, 35, 37, 43]. Dense connections involve the cerebello-thalamo-cortical motor pathways but are also found with prefrontal, cingulate, parietal, temporal and occipital cortex [8, 27, 28, 43]. It is increasingly recognised that most patients with chronic cerebellar degeneration develop neuropsychological symptoms [1, 14, 41]. Functional connectivity MRI (fcMRI) has revealed network alterations, which involve cerebellar and cerebral cortical areas in SCA2 [18, 26]. Also, other SCA subtypes such as SCA3 or SCA6 show patterns of neurodegeneration, which extend beyond the cerebellum and brainstem [16, 29, 32, 40]. The findings of extensive degeneration are also supported by neuropathological post-mortem findings in SCA2 and 6 [5, 7]. Given the strong evidence for intensive cerebello-cerebral interactions, it seems plausible that high-resolution structural imaging in cerebellar ataxia could also detect structural brain changes in distant areas of the cerebral cortex [22, 31]. Using fcMRI, the disruption of cerebello-cerebral networks can be detected. In addition, voxel-based-(vbm) and surface-based morphometry (sbm) could provide direct in vivo evidence for cerebral volume loss in chronic cerebellar degeneration, i.e. a distinction between functional alteration due to cerebellar network dysfunction and manifest cortical structural changes. Compared to SCA and SAOA, the symptomatology is rather restricted in patients with downbeat nystagmus syndrome (DBN) which report mainly oscillopsia and gait unsteadiness [31]. DBN phenomenology and its underlying structural correlates are thus putatively restricted to cerebellar regions of eye movement control and sensory integration [10, 12].

In the current study, we aimed to evaluate (i) a possible differential pattern of volume loss in patients with DBN syndrome, SAOA and SCA, (ii) the extent and pattern of distant volumetric changes in the cerebral cortex and sub-cortex and (iii) the association of structural degeneration with functional disability in the motor and vestibular system and locomotion.

## Methods

### Patients

We studied 30 patients with cerebellar degeneration of different aetiologies that were referred to the *German Center for Vertigo and Balance Disorders* of the Munich university hospital, LMU Munich between 2018 and 2020. Of those, 14 patients had downbeat nystagmus syndrome (DBN), seven patients had sporadic adult-onset ataxia (SAOA) and nine patients had spinocerebellar ataxia (SCA2: *n* = 6; SCA3, 6, 28: *n* = 1 each). Genetic testing was performed in patients with spinocerebellar ataxia to confirm the diagnosis in advance (pathological CAG repeat expansions were found in the ATXN2 gene in SCA2, in the ATXN3 gene in SCA3, in the CACNA1A gene in SCA6, and a pathological variant in the AFG3L2 gene in SCA28). Each patient received a full aetiological workup which included CSF analysis and antibody testing. In the setting of a tertiary specialised centre for vertigo and ocular–motor disorders, most of the time, this workup was completed before patients came to our attention. Patients with cerebellar degeneration with insidious onset and/or additional signs of peripheral neuropathy and vestibulopathy were tested for RFC1 gene mutations. In our sample, none of the patients were GAD-antibody positive and no patients presented with the triad of cerebellar syndrome, peripheral neuropathy and vestibulopathy. There was no case of RFC1 gene mutation. Patients were classified as SAOA or DBN after exclusion of inflammatory, metabolic, vascular, morphological, paraneoplastic, autoimmunological, familial cerebellar ataxia and toxic/pharmacological aetiologies.

### Controls

A group of 29 healthy age- and gender-matched healthy controls (HC) with no history of acute or chronic neurological disease received the identical imaging protocol for group comparisons.

### Clinical and fall-risk assessment

An assessment of the ambulatory status, disease duration and comorbidities was carried out with all participants in a standardised interview. Falls were assessed retrospectively containing information on fall status (yes or no), and subjective stability was examined by the Falls Efficacy Scale-International (FES-I) [44]. The cognitive performance was measured with an established screening tool (Montreal cognitive assessment, MoCA), the global severity of ataxic symptoms with the scale for the assessment and rating of ataxia (SARA) [23, 30]. Gait performance was characterised by preferred walking speed assessed on a pressure-sensitive gait carpet (GAITRite, CIR System, Sparta, NJ, USA).

A standardised physical and neurological examination was carried out with each participant. Furthermore, all patients received standardised neuro-orthoptic testing (spontaneous eye position and movements (nystagmus), static otolith function using the subjective visual vertical (SVV), optokinetic nystagmus, vestibulo-ocular reflex (VOR), saccades, smooth pursuit). For the SVV, a mean deviation of >  ± 2.5° over seven binocular measurements was considered pathological.

Symptomatic medication details were recorded for all cerebellar degeneration patients. In addition to professional physiotherapy (usually one 1-h session per week), all patients were instructed to complete a daily exercise and balance training programme. All patients received identical recommendations for professional and self-supervised physiotherapy.

### Imaging

All patients and HC received high-resolution structural MR-imaging on a clinical 3 T MRI scanner (T1 MPRAGE, 0.75 mm^3^ isotropic, 320 slices, TR 2060 ms, TE 2.17 ms, Magnetom Skyra, Siemens Healthcare, Erlangen, Germany).

#### Voxel-based morphometry (VBM)

We used the CAT12 toolbox version 1739 (Gaser & Dahnke, Department of Psychiatry, University of Jena, Jena, Germany; http://www.neuro.uni-jena.de/cat) within Statistical Parametric Mapping SPM12, version 7771 (https://www.fil.ion.ucl.ac.uk/spm/; Wellcome Department of Cognitive Neurology), using Matlab R2019b (Mathworks) for data quality estimation, preprocessing, and analysis of the data after standard preprocessing, applying a 6 mm Gaussian smoothing kernel. The modulated grey matter (GM) and white matter (WM) images were used for the volumetric analysis.

#### Surface-based analyses (SBM)

The CAT12 toolbox contains a fully implemented and validated processing pipeline for SBM [13]. We analysed distinct parameters of the cortical geometrical surface, such as cortical thickness (CT), sulcal depth (SD), the fractal dimension (FD) and gyrification indices (GI), to evaluate the complexity of the cortical surface based on the absolute mean curvature approach [17]. This allowed us to differentiate between cortical atrophy and changes of the cortical surface that are due to reshaping of the cortex in response to underlying white matter degeneration or the combination of both. For this purpose, the T1-weighted images underwent tissue segmentation to estimate white matter distance. Local maxima are projected to other grey matter voxels using a neighbour relationship, described by the distance to the white matter which equal cortical thickness. Partial volume correction, sulcal blurring and sulcal asymmetries without reconstruction of the sulci were applied. Topological correction is based on spherical harmonics. For the analysis, spherical mapping of the cortical surface is included [3, 46]. An adapted volume-based diffeomorphic DARTEL algorithm was then applied to the surface for spherical registration. Central cortical surfaces were created for both hemispheres separately. Surface reconstructions of the cortical values for each hemisphere were resampled to the 164 k mesh template space (*Freesurfer*) after merging and then smoothed with a 15 mm (cortical thickness), 20 mm (sulcus depth) and 23 mm (fractal dimension and gyrification index) Gaussian filter respectively.

### Statistical analysis

#### Group comparison and commonalities of cerebellar degeneration

Patients with SCA, SAOA or DBN were compared to the HC in an ANOVA model with group as a factor. Total intracranial volume (TIV) was modelled as a covariate of no interest for the volumetric analyses. Group level *t* statistics were performed for all possible pairs of between-groups comparison.

#### Association of clinical deficits with chronic cerebellar degeneration

All patients were included in a linear regression model using TIV and age as covariates of no interest. Variables of interest were the SARA, FES-I, MoCA score and the preferred gait velocity.

Specific clinical deficits, such as a history of falls, gaze-evoked nystagmus, and pathological tilts of the SVV (as dichotomous variables), were evaluated using two-sample t tests.

#### Correction for multiple comparisons

All imaging results from the ANOVA, *t* tests and linear regression models underwent extensive non-parametric permutation testing (*threshold-free cluster enhancement*, *TFCE*) with 10,000 permutations [34]. The results were corrected for multiple comparisons on the cluster level using family-wise error (FWE) correction. Results below a threshold of *p* < 0.05 were considered robust against false positives.

#### Clinical variables of interest

Distribution, frequency and correlations between the clinical variables of interest were analysed using SPSS (version 26.0.0.1; IBM Corp. 2019, Armonk, NY). Descriptive statistics are reported as mean ± SD or median ± IQR where applicable. First symptoms were recorded as those that led to neurological consultation. If more than one symptom was present, all symptoms at the time of presentation were recorded. Falls were defined as falling to the ground or to a lower level. An analysis of variance (ANOVA) was used to compare mean values of metric and categorial variables between the three patient groups. Tukey post hoc analysis was performed to identify significant differences between the groups.

#### Patient consent and data availability

The study was performed in accordance with the 1964 Declaration of Helsinki (latest applicable revision Fortaleza 2013) and approved by the institutional review board of LMU, Munich, Germany (no. 333-07). All patients gave informed written consent to participate in the study. The structural imaging patient data is not publicly available due to European Privacy laws and lack of consent for data sharing by the patients.

## Results

### Demographic and clinical data

Patients with DBN presented with vertigo (79% vs. 29% and 22%) and ocular–motor symptoms (43% vs. 14%, 0%), whereas gait instability was one of the first symptoms (100% vs. 29% in patients with DBN) in SCA and SAOA. A relevant proportion of patients with SAOA and SCA reported falls (71% and 89% respectively). SARA scores were significantly higher in patients with SCA and SAOA compared to patients with DBN (mean 11 points in the SAOA group and 10 points in the SCA group vs. 5 in the DBN group (*p* = 0.006). Balance confidence as assessed by the FES-I was lower in patients with SAOA and SCA compared to those with DBN (mean scores: 37 and 31 vs. 28). Only one patient had clinical signs of corticospinal tract involvement (spasticity, pathological reflexes, aetiology: SCA28). Preferred gait velocity showed no significant differences amongst the three patient groups. Cognitive function and static otolith function (SVV) were similar across all three diagnosis groups and mildly impaired in most patients (Table 1).

**Table 1:** Demographic and clinical data

Group	SAOA	SCA	DBN	HC	All	ANOVA *F*(3;47)	*p*
Demographic							
*n* (f/m)	7 (3/4)	9 (5/4)	14 (10/4)	21 (13/8)	51 (31/20)		
Age (years; mean ± SD)	59 ± 13	40 ± 17*	65 ± 11*	58 ± 21*,**	57 ± 19	4.3	0.010

Group	SAOA	SCA	DBN	All	ANOVA *F*(2;29)	*p*
Clinical characteristics						
First symptoms						
Gait disorder (%)	100	100	29	76.3		
Balance disorder (%)	43	11	14	22.7		
Ocular–motor/double vision (%)	14	0	43	19		
Vertigo (%)	29	22	79	43.3		
Speech disorder (%)	14	33	0	15.7		
Ataxia (%)	29	44	0	24.3		
Falls at disease onset (*n*)	71	89	29	63		
Disease duration (years; median, IQR)	6 ± 6	5 ± 6	6 ± 6	6 ± 5		
MOCA score (median, IQR)	26 ± 4	27 ± 4	27 ± 3	26 ± 3		
SARA score (mean ± SD)	11 ± 4*	10 ± 6*	5 ± 3*	8 ± 5	6.1	0.006
FES-I score (mean ± SD)	37 ± 12	31 ± 10	28 ± 7	31 ± 10	2	n.s
Preferred gait velocity (m/s; mean ± SD)	1.1 ± 0.2	0.9 ± 0.3	1.0 ± 0.3	1.0 ± 0.3	0.8	n.s
SVV tilts (°, mean ± SD)	2.9 ± 2.9	2.2 ± 1.9	1.9 ± 1.4	2.2 ± 2.0	0.6	n.s

*Significant difference in the Tukey post hoc comparison (DBN compared to SAOA and SCA)

**Healthy subjects were age-matched to each cerebellar degeneration group for the imaging analysis

Amongst the group of patients with DBN, four patients were already treated with 4-aminopyridine when the study was performed, three of them reported a subjective improvement of symptoms, and one stopped the medication because no improvement was noted. In five other patients, 4-aminopyridine treatment was started during the study period, in two patients we initiated treatment with acetyl-dl-leucine.

From the SAOA patient group, one patient was successfully treated with acetyl-dl-leucine, three patients showed no improvement of symptoms under both acetyl-dl-leucine and 4-aminopyridine therapy.

Of the patients with SCA, four patients with SCA2 and one patient with SCA28 responded well to acetyl-dl-leucine; one patient with SCA 6 did not improve with either acetyl-dl-leucine or 4-aminopyridine therapy.

### Partial correlations

We used partial correlations to evaluate associations between the variables of interest (disease duration, SARA score, FES-I score, tilts of the SVV, MoCA scores, gait velocity) using TIV and age as covariates of no interest (Table 2). Here, disease duration as an independent factor was not correlated with any of the variables. We found negative correlations between the variables SARA scores and MoCA scores (degrees of freedom: 26, *r*: − 0.5, *p* 0.007), the self-determined ambulation speed (*df* 26, *r* − 0.469, *p* 0.012), and the subjective fall efficacy (*df* 26, *r* 0.646, *p* < 0.0001). A positive correlation was found between the MoCA score and the preferred ambulation speed (*df* 36, *r* 0.595, *p* 0.001).

**Table 2:** Partial correlations of the variables of interest using TIV and age as covariates in all cerebellar patients (*n* = 30, 26 degrees of freedom)

	Symptom duration	SARA	MoCA	SVV tilt	preferred walking speed	FES-I
Symptom duration						
*r*	1.000	− 0.006	0.086	− 0.249	− 0.050	0.073
*p*		0.976	0.664	0.201	0.799	0.711
SARA						
*r*	− 0.006	1.000	**− 0.500**	0.335	**− 0.469**	**0.646**
*p*	0.976		**0.007**	0.081	**0.012**	**< 0.0001**
MoCA						
*r*	0.086	**− 0.500**	1.000	− 0.117	**0.595**	− 0.277
*p*	0.664	**0.007**		0.554	**0.001**	0.154
SVV tilt						
*r*	− 0.249	0.335	− 0.117	1.000	0.130	0.311
*p*	0.201	0.081	0.554		0.510	0.108
Preferred walking speed						
*r*	− 0.050	**− 0.469**	**0.595**	0.130	1.000	− 0.225
*p*	0.799	**0.012**	**0.001**	0.510		0.249
FES-I						
*r*	0.073	**0.646**	− 0.277	0.311	− 0.225	1.000
*p*	0.711	**< 0.001**	0.154	0.108	0.249	

*p < 0.05; two-sided

### Imaging

#### Structural whole brain changes in DBN, SCA and SAOA compared toHC

##### Voxel-based morphometry (VBM)

**Fig. 1:**
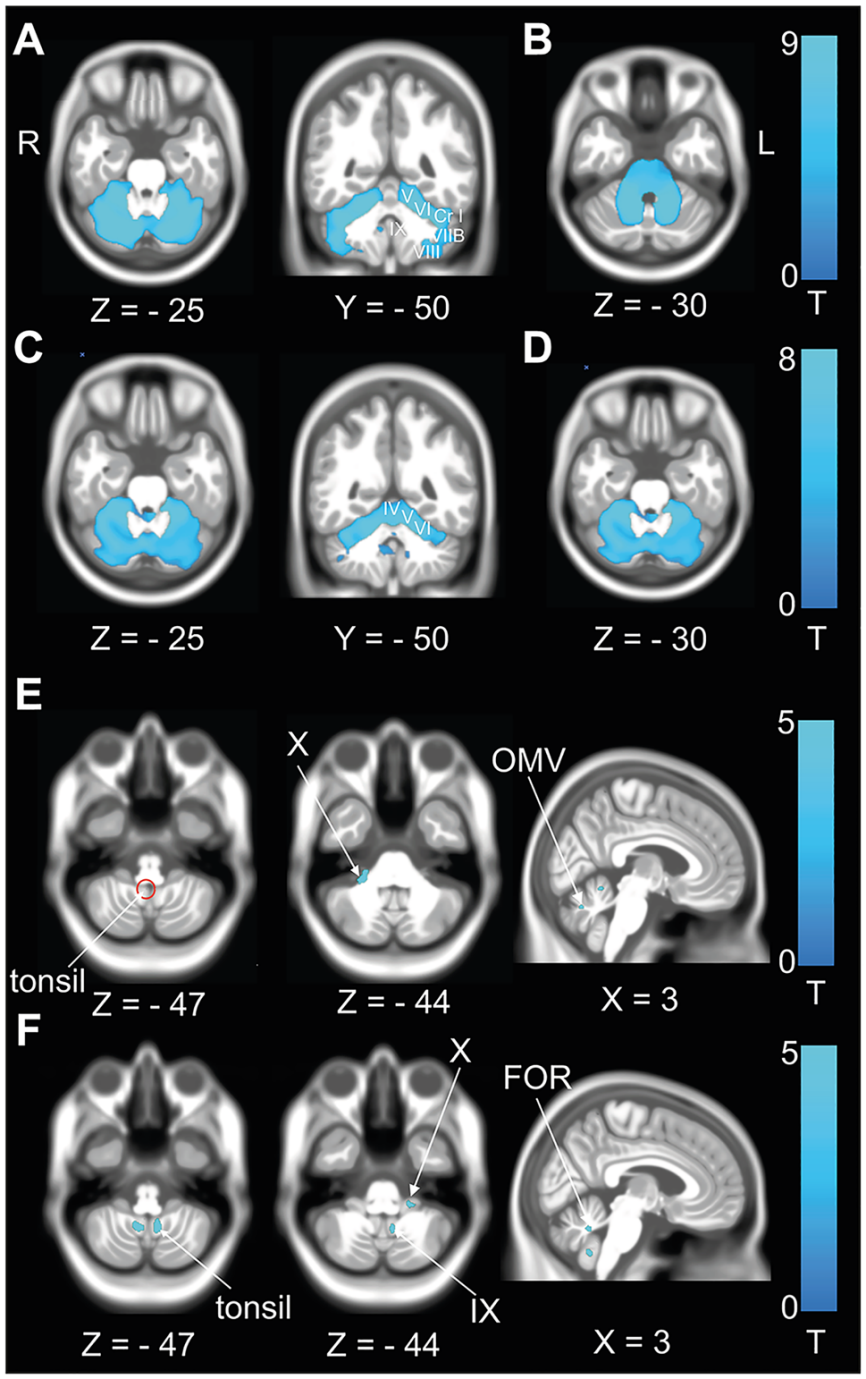
Differential patterns of volume loss in SCA, SAOA and DBN. **A** GMV reduction in patients with SCA compared to the HC. GMV reduction was detected in the motor cerebellum but extended in the hemispheres towards lobule VIIB, VIII and Crus I** B** WMV reduction in patients with SCA compared to HC. WMV reduction was detected in all three cerebellar peduncles and the adjacent pontine white matter up to the mesencephalon and descending to the medulla oblongata. **C** GMV reduction in patients with SAOA compared to the HC. Patients with SAOA also showed a widely distributed pattern of volume reduction. Compared to the patients with SCA, the pattern was more restricted to the motor lobules (IV–VI) and the vestibular (lobules IX, X) and ocular–motor cerebellar areas (OMV).** D** WMV reduction in patients with SAOA compared to the HC. Like patients with SCA, those with SAOA showed volume loss in the cerebellar peduncles and pontine white matter. All results after permutation testing using TFCE (10,000 permutations), *FWE* corrected *p* < 0.05. **E** GMV reduction in patients with DBN compared to HC. A GMV reduction was present in lobule X, the OMV (vermal lobules V-VII), nodulus and uvula (lobule IX) and the cerebellar tonsils representing the key cerebellar structures for ocular–motor and vestibular processing. An additional cluster was found in hemispheric lobules I–IV.** F** WMV reductions in patients with DBN compared to HC was observed in corresponding parts of the white matter in flocculus, fastigial oculomotor region (FOR), and lobule IX (nodulus/uvula) (*p* < 0.001, uncorrected)

*Grey matter volume (GMV) in the cerebellum* GMV was reduced in the cerebellar cortex in SCA and SAOA compared to the HC. A more prominent GMV reduction in SCA was found in the cerebellar hemispheres (lobules V, VI, Crus I, VII, and VIII) compared to the midline structures (Fig. 1A). In patients with SAOA, GMV reductions were more restricted to the motor (lobule IV-VI) and midline cerebellar structures (lobules IX, vermal V–VII, ocular–motor vermis (OMV)) (Fig. 1C). Patients with DBN syndrome showed a GMV reduction restricted to the flocculus (lobule X), OMV (lobules V–VII) and nodulus/uvula (lobule IX) (Fig. 1E).

*White matter volume (WMV) in the cerebellum* Corresponding to the GMV reductions, patients with SCA and SAOA showed extensive WMV loss in the cerebellar hemispheres and vermis. These WMV reductions extended to the brainstem in the patients with SCA, likely demonstrating volume loss in the cerebellar peduncles up to the midbrain (Fig. 1B, D). We found WMV reductions in the flocculus (lobule X), the fastigial oculomotor region (FOR), nodulus /uvula (lobule IX) and the cerebellar tonsils in patients with DBN compared to HC (Fig. 1F).

##### Voxel-based morphometry—SARA scores

**Fig. 2:**
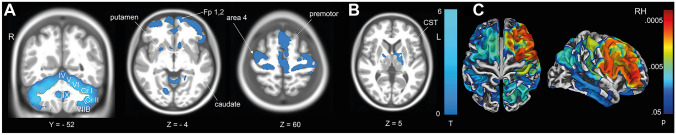
Volumetric decreases associated with disease severity measured with total SARA score. Linear regression: SARA scores in all cerebellar degeneration patients. **A** Negative correlation of GMV with higher total SARA scores. Significant voxels are located in the cerebellum, basal ganglia (putamen, caudate), primary motor cortex (area 4) and prefrontal cortex (Fp1,2). **B** Negative correlation of WMV with the SARA score reached the corticospinal tract (CST). **C** Negative correlation of cortical thickness with the SARA score were most prominent in the premotor cortex and dorsolateral prefrontal cortex (DLPFC). All vbm results after permutation testing using TFCE (10,000 permutations), *FWE* corrected *p* < 0.05. All sbm results are depicted with logarithmic *p* value scales FWE corrected after TFCE (10,000 permutations)

*GMV* The regression model using the total SARA score showed a negative correlation with clusters in the cerebellum (lobules IV–VI, Crus I, II, VIIB, IX), the basal ganglia (putamen and caudate nucleus) and also prefrontal cortex (areas Fp1,2) and (pre-)motor cortex (areas 4,6) (Fig. 2A).

*WMV* The total SARA score showed a negative correlation with clusters in the cerebellar white matter, brainstem and reached the corticospinal tract (Fig. 2B).

##### Surface-based morphometry—SARA score

*Cortical thickness* In congruence with the volumetric findings, we found a negative correlation of CT with higher SARA scores. This effect was most prominent in the right prefrontal cortex but also included the left prefrontal as well as the temporal and parietal lobes. (Fig. 2C). No changes in SD, GI or FD were observed.

##### Voxel-based morphometry—Falls efficacy scale (FES-I)

**Fig. 3:**
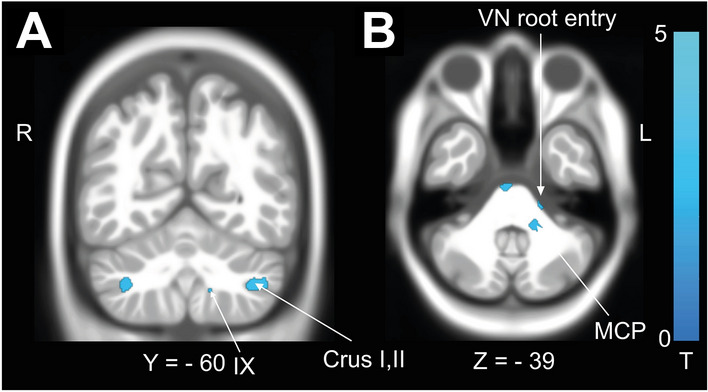
Volumetric changes associated with subjective postural stability (FES-I). Subjective postural stability was correlated with **A** GMV in cerebellar lobules Crus I, II and **B** WMV in the middle cerebellar peduncle and around the vestibular nerve (VN) root entry zone. All results after permutation testing using TFCE (10,000 permutations), *FWE* corrected *p* < 0.05

*GMV* Balance confidence as assessed by the FES-I was negatively correlated with the integrity of cerebellar lobules Crus I, II and lobule IX. No association with cortical GMV was found for subjective postural stability. (Fig. 3A).

*WMV *We found a negative correlation of WMV in the middle cerebellar peduncle and around the vestibular nerve root entry zone with the FES-I scores (Fig. 3B).

##### Voxel-based morphometry—MoCA scores

*GMV* The GMV reduction in relation to the MoCA score was remarkably similar compared to those of the SARA score which reflects the correlation between the variables (positive correlation of the MoCA score with brain volume). However, some differences have to be noted. Compared to the SARA score, volumetric changes in the cerebellum were restricted to lobules Crus I, II, lobule IX, dentate nucleus for the MoCA score.

**Fig. 4:**
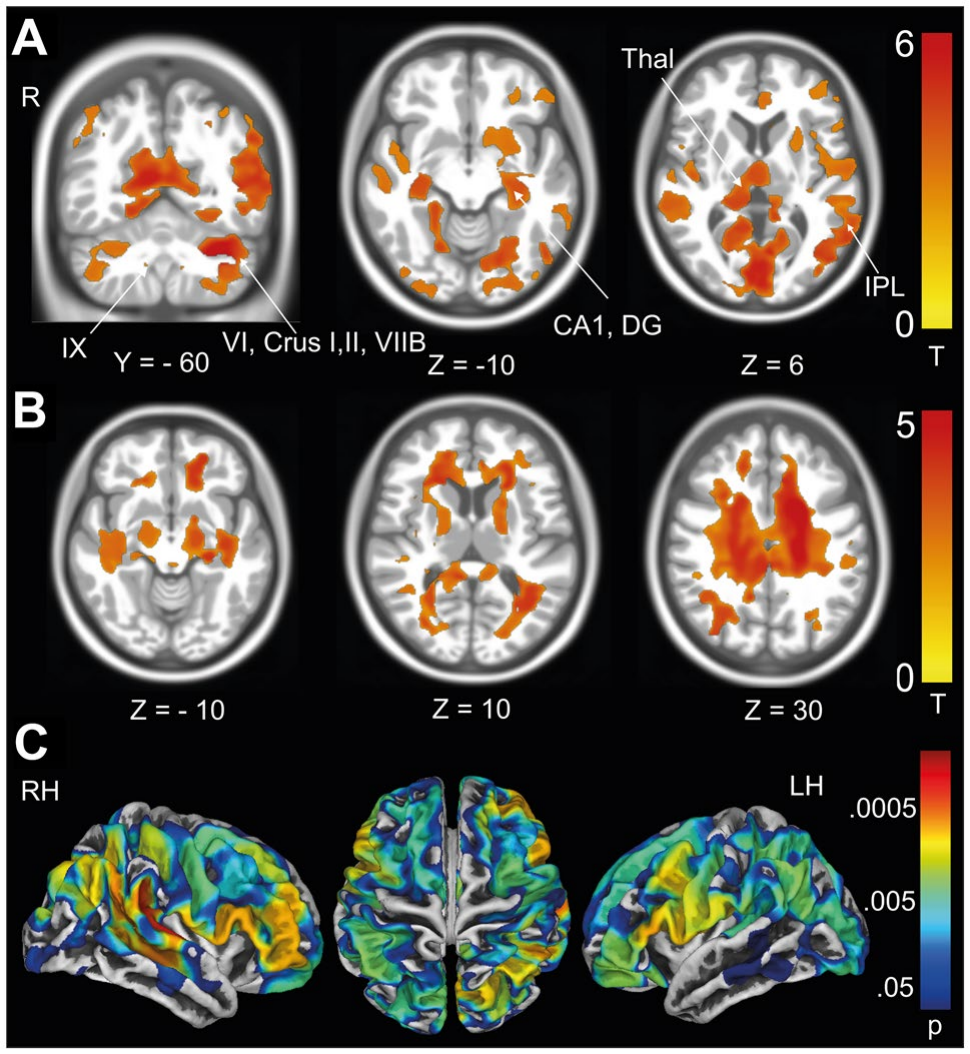
Volumetric changes associated with cognitive performance (MoCA score). **A** A higher MoCA score was associated with GMV in the cerebellar cognitive lobules Crus (Cr) I, II, lobule IX, the thalamus, the hippocampus (areas CA1,DG (dentate gyrus)), inferior parietal lobule (IPL), but also prefrontal, orbitofrontal and premotor cortex. **B** A correlation of WMV with the MoCA score was detected around the hippocampi, and along the anterior thalamic radiation, but also in the white matter of the premotor cortex. **C** *SBM* showed a correlation of cortical thickness with dorsolateral prefrontal and parietal cortex. *VBM* results are thresholded at *p* < 0.05, *FWE* corrected for multiple comparisons on the cluster levels using *TFCE* (10,000 permutations). *SBM* overlays are depicted with logarithmic p-value scales *FWE* corrected after *TFCE* (10,000 permutations)

In the (sub-)cortex, volumetric changes associated with the MoCA score involved the thalamus, the hippocampus, prefrontal and orbitofrontal cortex and the inferior parietal lobule as hubs of cognitive/default mode network hubs for the MoCA. Additional correlations were found with visual cortex and the subcortical and cortical motor network (Fig. 4A).

*WMV* The WMV changes associated with the MoCA score involved cerebello-thalamo-cortical connexions with the prefrontal and motor cortex, the hippocampus and also interhemispheric connections through the posterior segments of the corpus callosum (Fig. 4B).

##### Surface-based morphometry—MoCA score

Similar to the GMV changes, cortical thickness in the prefrontal, and temporo-parietal cortex was positively correlated with higher MoCA scores (Fig. 3C).

##### Voxel-based morphometry—preferred gait velocity GMV

**Fig. 5:**
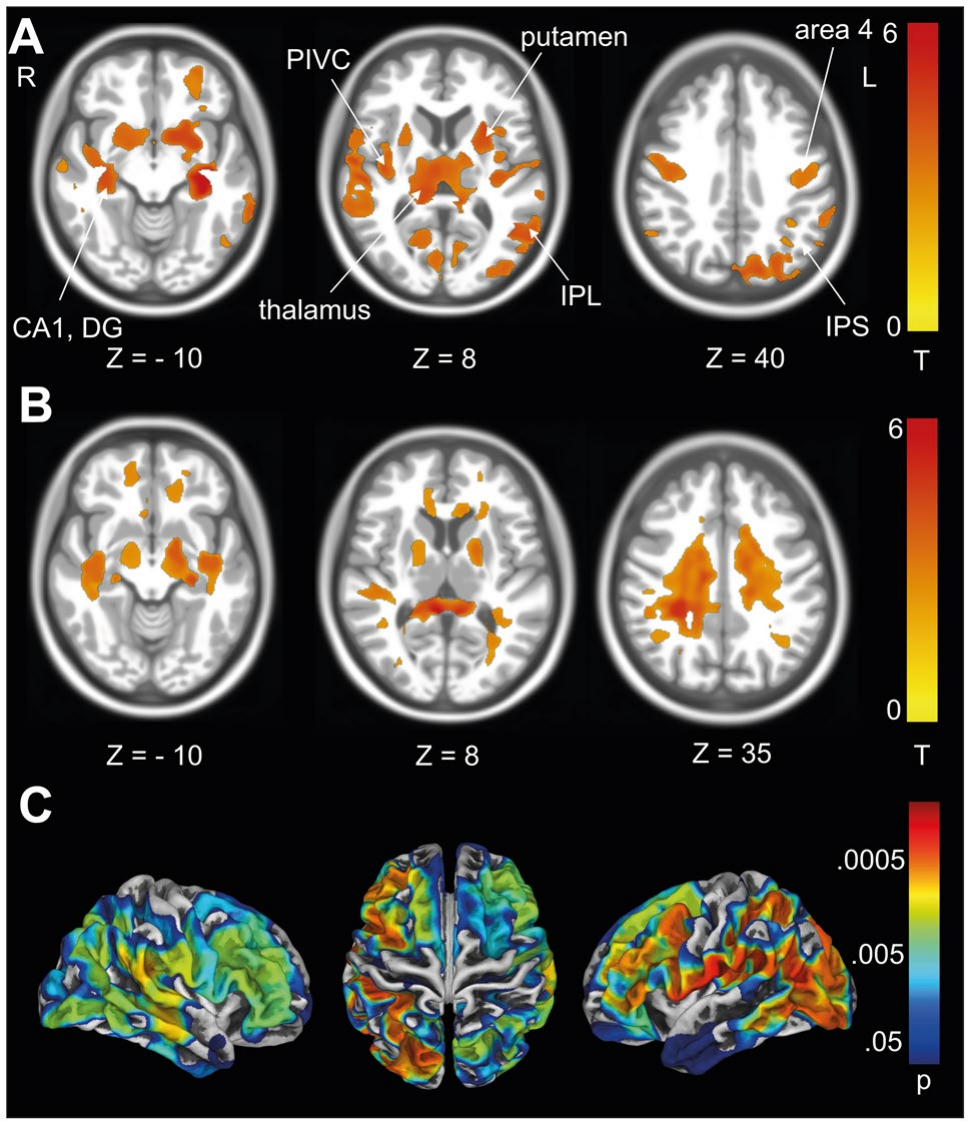
Volumetric associations with preferred walking speed. **A** An association of *GMV* was observed with multisensory integration centres for spatial orientation, navigation (PIVC, IPS, visual cortex, IPL, hippocampus (subfields CA1,2)) and in the primary motor network (putamen, area 4). **B** An association with white matter volume was observed with white matter tracts around the hippocampus and PIVC, the corticospinal tract and the corpus callosum. **C** *SBM* showed an association of cortical thickness with dorsolateral prefrontal, primary sensory–motor and parietal areas. *VBM* results after permutation testing using *TFCE* (10,000 permutations), *FWE* corrected *p* < 0.05. SBM overlays are depicted with logarithmic *p* value scales *FWE* corrected after TFCE (10,000 permutations)

An association of GMV with self-determined gait velocity was observed with multisensory integration centres for spatial orientation, navigation and cognition (PIVC, IPS, visual cortex, IPL, hippocampus (subfields CA1,2)) and in the primary motor network (putamen, area 4) (Fig. 5A).

*WMV* An association of preferred gait velocity with WMV was observed around the hippocampus and PIVC, the corticospinal tract and the corpus callosum (Fig. 5B).

##### Surface-based morphometry—preferred gait velocity

SBM showed an association of cortical thickness with dorsolateral prefrontal, primary sensory–motor and parietal areas (Fig. 5C).

##### Clinical deficits

Volumetric changes in patients with ocular–motor (gaze-evoked nystagmus) and vestibular perceptual deficits (pathologic tilts of SVV).

**Fig. 6:**
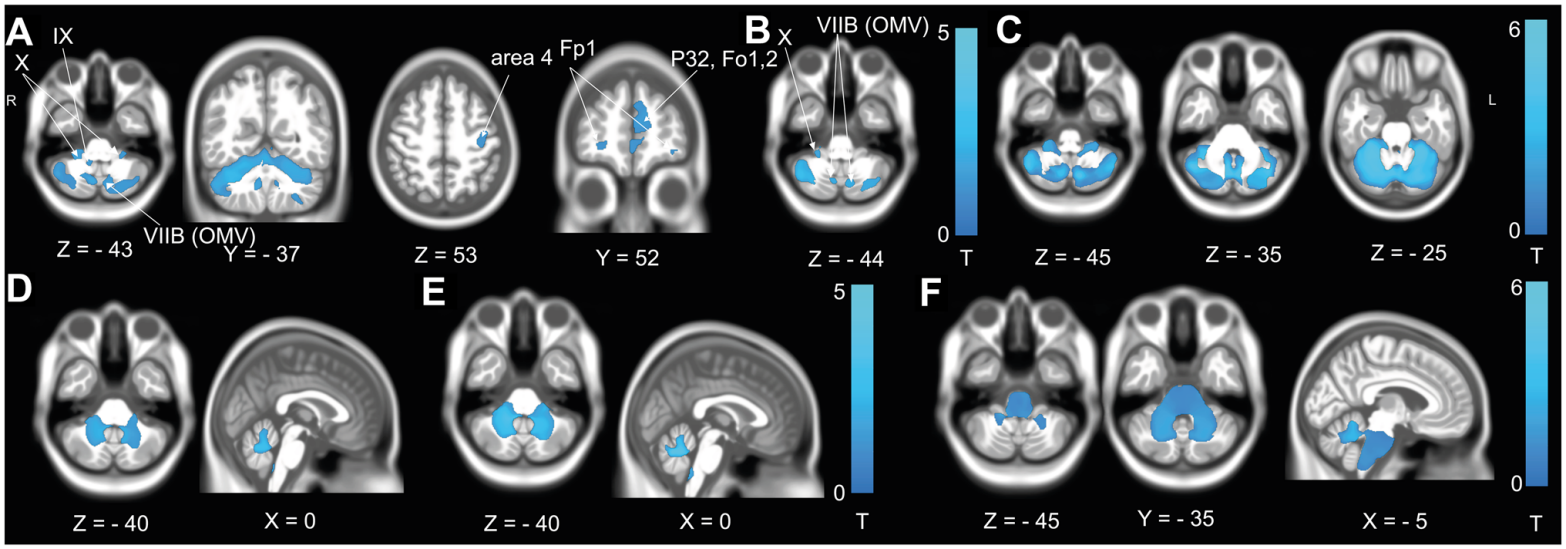
Volume loss in cerebellar degeneration related to ocular–motor deficits, vestibular perceptual deficits and the history of falls. **A** GMV reduction in patients with tilts of the SVV compared to the HC. Volume reductions were found in vestibular and ocular–motor cerebellar lobules (IX, X, VIIB, corresponding to nodulus, uvula, flocculus and ocular–motor vermis (OMV). Additional GMV loss was detected in the cerebellar hemispheres. Remote volume loss was also observed in primary motor (area 4) and rostral frontal cortex (frontal pole Fp1, orbitofrontal cortex Fo 1,2 and rostral cingulate cortex p32). **B** GMV reduction in patients with gaze-evoked nystagmus compared to the HC were also located in lobules X, OMV and the cerebellar hemispheres. **C** GMV reduction in patients with falls at disease onset was observed in all cerebellar lobules. WMV loss in patients with SVV tilts (**D**) and gaze-evoked nystagmus (**E**) compared to the HC was restricted to the cerebellum around the vestibular and ocular–motor regions. In contrast, patients with a history of falling (**F**) had additional WMV loss in the brainstem, likely reflecting volume loss in the cerebellar peduncles. All results after permutation testing using TFCE (10,000 permutations), *FWE* corrected *p* < 0.05

*GMV* Patients with pathological tilts of the SVV compared to those with no tilts (< 2.5°) of the SVV had GMV reduction in the key vestibulo-cerebellar hubs in lobules IX (nodulus, uvula) and X (flocculus) and lobule VIIB (OMV) and in the cerebellar hemispheres (lobules IV–VI, Crus I). Additional clusters of GMV reduction were observed in the cerebral hemispheres in primary motor cortex (area 4) and the frontal pole and orbitofrontal cortex (Fp1, P32, Fo1,2) (Fig. 6A).

Patients with gaze-evoked nystagmus showed volume loss restricted to the cerebellum (lobules X, VIIB) (Fig. 6B).

*WMV* In both patients with tilts of the SVV and patients with gaze-evoked nystagmus, WMV reduction was limited to the cerebellum and inferior cerebellar peduncle (Fig. 6D, E).

##### Volumetric changes in patients with falls at disease onset

*GMV* GMV reductions in patients with a history of falls was more extensive compared to those with ocular–motor or vestibular perceptual deficits. GMV reductions were restricted to the cerebellum (vermis and hemispheres) (Fig. 6C).

*WMV* In contrast to the patients with ocular–motor and vestibular perceptual deficits, WMV reduction in patients with a history of falls extended to the brainstem, likely by means of volume loss in the cerebellar peduncles (Fig. 6E).

## Discussion

In the current study, we found that: (I) Both SCA and SAOA led to widely distributed GMV and WMV loss in the cerebellar hemispheres. The WMV reduction extended to the brainstem and seems to be related to volume loss in the cerebellar peduncles. (II) In patients with DBN syndrome, volumetric changes follow the presumed pathophysiology with volume loss in the flocculus, nodulus, uvula and related white matter, not affecting other cerebellar functional modules or extra-cerebellar structures. (III) Falls are associated with volume loss in the brainstem. (IV) Functional disability is associated with distant volume loss in the cerebral cortex and sub-cortex.

### Imaging data

The current study extends previous findings of structural degeneration in different types of ataxias. In general, patients with SCA and with SAOA showed a wide distribution of cerebellar volume loss. In contrast, volume loss in DBN followed a more restricted pattern [12, 42]. Besides the GMV reduction in the main hubs for vestibular processing in the cerebellum, an additional GMV and WMV reduction was found in the OMV and FOR [12]. The specialisation of the OMV and FOR for horizontal saccade generation, adaptation and accuracy is well established [6, 24, 39, 45]. In lesions of the FOR or OMV, saccades are mainly reported to be horizontally deviated [36, 39]. Based on the volumetric findings, the OMV and FOR might also be involved in the vertical resetting after the involuntary upward drift in DBN.

### Structure–function association for ocular–motor/vestibular domain and falls

Both static otolith dysfunction (pathologic SVV tilts) and gaze-evoked nystagmus were associated with GMV and WMV reductions in the motor (lobules IV-VI) and vestibular/ocular–motor representations (lobules IX, X, OMV). SVV tilts were additionally associated with a GMV reduction in the primary motor cortex and prefrontal cortex, supporting the integrated nature of verticality processing [4].

Falls, as a hallmark of patients with cerebellar ataxia, were prevalent in our cohort. We found a widespread distribution of volume loss in the cerebellum and brainstem of fallers. Similarly, the regression models for balance confidence and fear of falling (i.e. FES-I) point towards a reduction of white matter connexions to the brainstem. Thus, falls would be the consequence of impaired structural connexions from the cerebellum to the brainstem for postural control, locomotion execution and vestibulo-spinal reflex functioning [11, 19].

### Structure–function association for walking speed and cognitive functions

The pattern of volumetric changes in the cerebral cortex and sub-cortex for these variables involved mainly sensorimotor integration centres for spatial orientation, navigation and cognition (PIVC, IPL, IPS, hippocampus), thus indicating an interrelationship of overall cognitive and locomotor functions to non-cerebellar integration sites of motion perception and sensorimotor processes.

### Structural correlates of ataxia severity

Using *VBM*, we found negative correlations between the SARA score and the cerebellum, brainstem, but also for multiple areas in the cortex and sub-cortex, such as the (pre-)motor cortex and frontal pole. Additionally, *SBM* revealed a negative correlation of cortical thickness with the SARA score, most strikingly in the premotor cortex and posterior parietal areas. Interestingly, these cerebral volumetric findings were also associated with preferred gait velocity and cognitive performance. The partial correlation analysis revealed relevant correlations between these measures. This suggests that the volumetric cerebello-cortical correlations may reflect overall functional disability in chronic cerebellar degeneration with respect to higher order processing (i.e. gait speed, cognitive processing capacity). Since these measures were not correlated with disease duration, longitudinal data might help to further investigate the effect of disease progression on structural brain findings. It is our interpretation that the atrophy patterns above reflect the network structure of cerebello-cortical processing.

Atrophy patterns in SCA are grossly classified as involving the cerebellum only (i.e. in our cohort SCA6), pontocerebellar atrophy (SCA3) or even the cortex (SCA2) [20–22]. Widely distributed neurodegeneration has also been reported in other types of SCA including SCA3 and SCA6 [7, 32]. Given the preponderance of patients with SCA2 and SAOA in our sample, the *VBM* and *SBM* findings are likely driven by these patients.

On the other hand, DBN seems to be a very confined ocular–motor/vestibulo-cerebellar system degeneration.

In summary, the current findings indicate a phenomenology-dependent structural degeneration of different types of cerebellar disorders. Most strikingly, DBN appears to be a degeneration of mainly midline, vestibular– and ocular–motor cerebellar hubs. The challenging health problem of falls in cerebellar ataxia seems to be related to impaired connexions to the brainstem. Moreover, extra-cerebellar degeneration is present in SCA and SAOA and associated with verticality perception, global functional (dis-)ability, such as gait speed and cognitive functioning, as well as the overall severity of ataxia.

### Limitations

Our patient sample includes a variety of causes of cerebellar degeneration with a moderate overall sample size. The latter is due to the rarity of cerebellar degeneration patients even in the setting of a tertiary, research-oriented facility. Furthermore, we refrained to test correlations of ataxia subtypes with whole brain imaging, given the sample size. Therefore, with the current data, functional disability in distinct subtypes of cerebellar degeneration cannot be correlated with whole brain volumetry.

However, as neurodegeneration has been shown to extend beyond the boundaries of the historical descriptions of these entities, the aim of this study was to establish common principles of structural brain changes in relation to clinical deficits in cerebellar degeneration in general. We were particularly interested in cerebello-cortical interactions.

Differential changes in functional connectivity have been established for different SCA subtypes based on resting-state fMRI [25, 26, 40]. We did not deem it appropriate to discuss the differential pattern of volumetric changes in comparison to fMRI findings in distinct SCA aetiologies due to the differences in the group composition and the methodological differences.

More recently, standardised evaluation tools have been presented to grasp differential aspects of cerebellar dysfunction, including the cognitive (CCAS scale) and ocular–motor domains (SODA score) [9, 33]. In the current study, we were particularly interested in the effects of cerebellar degeneration on cortical structure and function. Therefore, we used the Montreal Cognitive assessment as a tool to screen for cognitive dysfunction which is validated for extra-cerebellar neurodegeneration. It should be noted in addition that the CCAS scale and the MoCA cover similar functional domains.

To thoroughly characterise the ocular–motor profiles of our sample, all patients received a dedicated neuro-orthoptic and video-oculographic evaluation which also included the estimation of the SVV and fundus photography. The use of an extensive quantitative ocular–motor examination paradigm likely sufficiently covers all aspects of the standardised SODA score.

We used meticulous clinical testing with an established quantitative evaluation of ataxia, subjective stability, vestibular–/ocular–motor function and walking performance to evaluate common and distinct patterns of volume loss in cerebellar degeneration. Extensive permutation testing and conservative *FWE* correction for multiple testing (robust against false-positive) were applied to account for the above-mentioned limitations.

## Conclusions

This study shows distinct volumetric changes in different types of cerebellar degeneration. Volumetric changes in cortical and subcortical hubs for motor performance, locomotion, navigation and cognition reflect functional disability in chronic cerebellar degeneration.
